# Superhydrophobic poly(L-lactic acid) surface as potential bacterial colonization substrate

**DOI:** 10.1186/2191-0855-1-34

**Published:** 2011-10-22

**Authors:** Cláudia Sousa, Diana Rodrigues, Rosário Oliveira, Wenlong Song, João F Mano, Joana Azeredo

**Affiliations:** 1 Institute for Biotechnology and Bioengineering, Centre of Biological Engineering, University of Minho, Campus de Gualtar, 4710-057 Braga, Portugal; 2 3Bs Research Group - Biomaterials, Biodegradables and Biomimetrics, AvePark, Zona Industrial da Gandra, S. Cláudio do Barco, 4860-909 Caldas das Taipas, Guimarâeas, Portugal; 3 ICVS/3B's - PT Government Associate Laboratory, Braga/Guimarâeas, Portugal

**Keywords:** Poly(L-lactic acid), Superhydrophobicity, Biomimetic surfaces, Bacterial colonization substrate

## Abstract

Hydrophobicity is a very important surface property and there is a growing interest in the production and characterization of superhydrophobic surfaces. Accordingly, it was recently shown how to obtain a superhydrophobic surface using a simple and cost-effective method on a polymer named poly(L-lactic acid) (PLLA). To evaluate the ability of such material as a substrate for bacterial colonization, this work assessed the capability of different bacteria to colonize a biomimetic rough superhydrophobic (SH) PLLA surface and also a smooth hydrophobic (H) one. The interaction between these surfaces and bacteria with different morphologies and cell walls was studied using one strain of *Staphylococcus aureus *and one of *Pseudomonas aeruginosa*. Results showed that both bacterial strains colonized the surfaces tested, although significantly higher numbers of *S. aureus *cells were found on SH surfaces comparing to H ones. Moreover, scanning electron microscopy images showed an extracellular matrix produced by *P. aeruginosa *on SH PLLA surfaces, indicating that this bacterium is able to form a biofilm on such substratum. Bacterial removal through lotus leaf effect was also tested, being more efficient on H coupons than on SH PLLA ones. Overall, the results showed that SH PLLA surfaces can be used as a substrate for bacterial colonization and, thus, have an exceptional potential for biotechnology applications.

## Introduction

Industrial bioconversion processes can be performed using different kinds of reactors, some of which are called "immobilized cell reactors", (Tyagi and Ghose 1982) that imply high cell concentrations, normally achieved by fixing the cells on various substrates. Adsorption is one of the different techniques used to immobilize microbial cells, rendering the immobilization process more economic and the reactors simpler in concept and construction. In fact, it is a natural immobilization process, since cells adsorb and adhere to the support naturally and firmly (Tyagi and Ghose 1982; Forberg and Haggstrom 1985; Qureshi and Maddox 1987), eventually developing into biofilms. On the other hand, surface has an important impact on bacterial colonization and several different materials have been used as substrata for cell immobilization, such as rocks, sands, latex, and steel. There are several criteria used to characterize a good substratum, and surface characteristics suitable for bacterial attachment must definitely be taken into consideration. Among surface physicochemical properties, hydrophobicity has been considered one of the most important, since in biological systems hydrophobic interactions are the strongest long-range non-covalent interactions, being considered a determining factor in microbial adhesion to surfaces (Sanin et al. 2003). Moreover, it has been shown that biofilm formation tends to increase with the hydrophobicity of the surface material (Donlan and Costerton 2002).

Since artificial superhydrophobic surfaces were first demonstrated in the mid-1990s (Onda et al. 1996), a very large number of inventive ways to produce rough surfaces that exhibit superhydrophobicity have been reported. Accordingly, a great deal of research has been devoted to the preparation and theoretical modelling of superhydrophobic surfaces (Nakajima et al. 2001; Callies and Quéré 2005; Parkin and Palgrave 2005; Sun et al. 2005), which result from the combination of a very large contact angle (≥ 150°) and a low contact-angle hysteresis. This kind of materials was originally inspired by the unique water-repellent properties of the lotus leaf (Barthlott and Neinhuis 1997) and the leaves of a number of other plants (Bhushan and Jung 2006). The so called "lotus effect", the superhydrophobicity of the surface, is the result of specific surface features on the lotus leaf: small nano-sized "bumps" make the contact surface area between the droplet and the leaf extremely small. This minimizes the attractive forces between the water molecules and the atoms of the surface, and allows the water to "bead up" and rolls off (Frim 2008).

Various methods have been proposed to fabricate superhydrophobic surfaces, such as the solution method (Erbil et al. 2003; Xie et al. 2004; Shi et al. 2008; Oliveira et al. 2010), the sol-gel method (Tadanaga et al. 2000; Shirtcliffe et al. 2003; Wang et al. 2005), solidification of alkylketene dimer (Onda et al. 1996), the plasma fluorination method (Teare et al. 2002; Woodward et al. 2003), among others (Bico et al. 1999; Nakajima et al. 1999; Genzer and Efimenko 2000; Nakajima et al, 2000). Poly(L-lactic acid) (PLLA) is a biodegradable polymer that has been used to produce superhydrophobic surfaces and has received substantial attention, not only due to its renewable resources (Tsuji et al. 1998) but also because of its biocompatibility, as well as excellent thermal and mechanical properties, and superior transparency of the processed materials (Urayama et al. 2002). In fact, biodegradable polymers have been receiving an increasing interest for biomedical applications, given that biodegradable polymeric films have potential applications for cell growth substrata, tissue engineering and drug delivery. In this context, Song et al. (2009) developed recently robust hydrophobic (H) and superhydrophobic (SH) PLLA substrates using a simple, cost-effective, and novel method. They have also studied the colonization of those materials by animal cells (mouse lung line L929) after different surface treatments. These authors observed that almost no animal cell adhesion occurred on the SH PLLA surfaces in comparison with the smooth (H) ones, and that the enhancement in hydrophilicity resulting from Ar-plasma treatment may greatly improve animal cell attachment. Similar behaviour was found with bone marrow derived cells on such kind of substrates (Alves et al. 2009). However, to our knowledge, there are no reports regarding bacterial interaction with such surfaces. Therefore, the present work aimed at studying the microbial colonization of H and SH PLLA surfaces by different kinds of bacteria and, consequently, evaluating the potential application of these materials as substrates in the biotechnological field when high levels of immobilized biomass are required.

## Materials and methods

### Poly(L-lactic) surfaces

The methodology used to obtain H and SH PLLA surfaces was the same previously described by Song et al. (2009). Briefly, a commercially available PLLA of high stereoregularity (Cargill Dow Polymer Mn = 69 000, M_w_/M_n _= 1.734) was converted to a flat rigid smooth PLLA substrate by melting the PLLA powder over a glass slide, compression with another glass slide, and further cooling in water. On the other hand, the rough SH surface was obtained using a PLLA/dioxane 13% (w/w) solution that was cast on the previous (smooth) substrate. The surface morphology of both kinds of PPLA surfaces was assessed through scanning electron microscopy (SEM), while the roughness of the superhydrophobic PLLA substrate was assessed using a NT1100-Optical Profiler.

Round shaped coupons with 2.5 mm in diameter of each type of PLLA surfaces were used to assess bacterial adhesion. The coupons were previously cleaned by immersion in a 70% ethanol solution, and then aseptically and individually washed with ultra-pure sterile water and let to dry overnight at room temperature (21°C).

### Bacterial strains and culture conditions

In order to assess the interaction between the distinct PLLA surfaces with different bacterial cell walls, this work included the Gram-positive *Staphylococcus aureus *CECT 239 (CECT, Colección Española de Cultivos Tipo), and the Gram-negative *Pseudomonas aeruginosa *ATCC 10145 (ATCC, American Type Collection Culture).

For each experiment, bacteria were subcultured on tryptic soy agar (TSA, Merck, Darmstadt, Germany) for about 36 h at 37°C and then grown for 24 h in 15 mL of tryptic soy broth (TSB, Merck), at 37°C under a constant agitation of 120 rpm (SI50; Stuart Scientific, Redhill, UK). After this period, an aliquot of 50 μL of the culture suspension was transferred into 30 mL of fresh TSB and incubated for 18 h under the same conditions in order to obtain a midexponential growth culture. Cells were harvested by centrifugation at 9000 rpm at 4°C for 5 minutes (3-16 K, Laborzentrifugen GmbH, Osterode, Germany) and washed twice with a saline solution [0.9% NaCl (w/v) (Merck) in sterile distilled water]. The cellular suspensions were adjusted to a final concentration of 1 × 10^8 ^cells per mL, determined by optical density at 640 nm, prior to subsequent assays.

### Surface colonization and cell enumeration

In order to promote bacterial colonization, each clean coupon of H and SH PLLA surfaces was placed into an individual well of a 24-well microtiter plate containing 1.5 mL of TSB enriched with 0.25% of glucose (Merck). For each bacterium, a 50 μL 1 × 10^8 ^cells/mL inoculum was added per well. The plates were incubated for 24 h at 37°C in an orbital shaker (120 rpm). All experiments were performed in triplicate, in three independent occasions.

After the incubation period, the number of bacterial cells colonizing the surfaces was determined by colony forming units (CFU) enumeration. In order to do so, coupons were transferred to a new 24-well microtiter plate and carefully washed twice with NaCl 0.9%. For each bacterium and material tested, four coupons were placed in a sterile eppendorf containing 1 mL of NaCl 0.9% and vortexed vigorously for 2 minutes. Next, the content of each eppendorf was sonicated twice (20 s, 22% of amplitude) (Ultrasonic Processor, Cole-Parmer, Illinois, USA) in order to detach the cells from the coupons. The remaining suspension was centrifuged (5 min, 9000 rpm, 4°C) and resuspended in 1 mL of NaCl 0.9%. Viable cells were determined by performing 10-fold serial dilutions in saline solution (NaCl 0.9%) and plated in TSA, in triplicate. Prior to colony enumeration, the plates were incubated for 24 h at 37°C.

### Bacteria removal assays

Bacteria removal assays were performed to assess the "self-cleaning" character of both kinds of PLLA surfaces. For that, the same experimental procedure described for the colonization assays (section 2.3) was used, except that, before transferring to eppendorfs for sonication, each coupon was gently immersed in ultrapure sterile water and then tilted to allow the liquid to flow over the surface. The remaining cells were collected and enumerated as described in section 2.3.

### Scanning Electron Microscopy (SEM)

In order to observe how the different bacterial cells were distributed on the surface of both kinds of PLLA surfaces, coupons representing each experimental condition were visualized under a scanning electron microscope. Therefore, after the 24 h incubation period, coupons were dehydrated by a 15 min immersion in solutions with increasing concentrations of ethanol up to 100% (vol/vol), having then been placed in a sealed desiccator. Morphological analysis was performed in an Ultra-high resolution Field Emission Gun Scanning Electron Microscopy (FEG-SEM), NOVA 200 Nano SEM, FEI Company. Secondary electron images were performed with an acceleration voltage of between 5 e 10 KV. Before morphological analyses, the samples were covered with a very thin film of Au-Pd (80-20 weight %) with 8 nm thickness, in a high resolution sputter coater, 208 HR Cressington Company.

### Statistical analysis

Data analysis was performed using the statistical program SPSS (Statistical Package for the Social Sciences). The results were compared using the non-parametric Mann-Whitney U-test at a 95% confidence level.

## Results

### Quantification of bacterial colonization and removal

**Figure 1:**
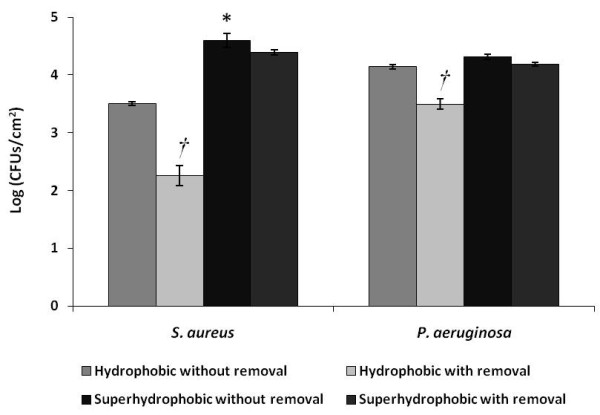
**Number of *S. aureus *and *P. aeruginosa *cells, per square centimetre of SH PLLA and H PLLA surfaces, after colonization and removal assays**. Symbols indicate statistically different values (p < 0.05) between colonization of both kinds of surface considering the same bacteria (*), and between the amount of cells present on a same surface before and after the removal procedure (*†*).

As can be seen in Figure 1, the enumeration of *S. aureus *and *P. aeruginosa *cells showed that both bacteria extensively colonized both PLLA surfaces, achieving values of 4 Log CFUs/cm^2^. Nevertheless, a significant higher amount of *S. aureus *cells was found on the SH surface comparing to the H one. Regarding H surface colonization by both bacteria, a significant greater amount of *P. aeruginosa *cells was found comparing to *S. aureus *(Figure 1).

As far as removal assays are concerned, it was observed that both bacteria did not suffer a significant decrease of biomass amount on the SH surface (Figure 1). In contrast, the removal was more effective on the H surface, since both bacteria suffered a significant reduction (1 Log reduction) on the number of cells adhered to this substratum comparing to the values found when no removal procedure was performed.

### Surface morphology and roughness and spatial distribution of bacterial cells

**Figure 2:**
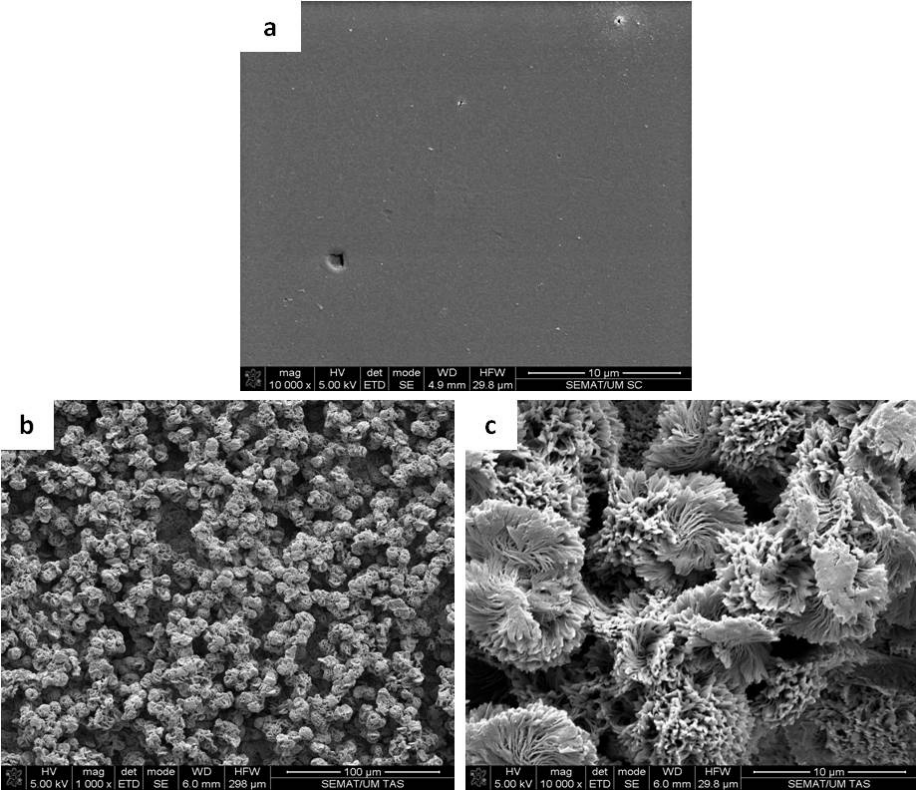
**SEM images of (a) the smooth surface of the H PLLA, (b) the rough surface of SH PLLA, and (c) the protrusions on the SH PLLA surface**.

**Figure 3:**
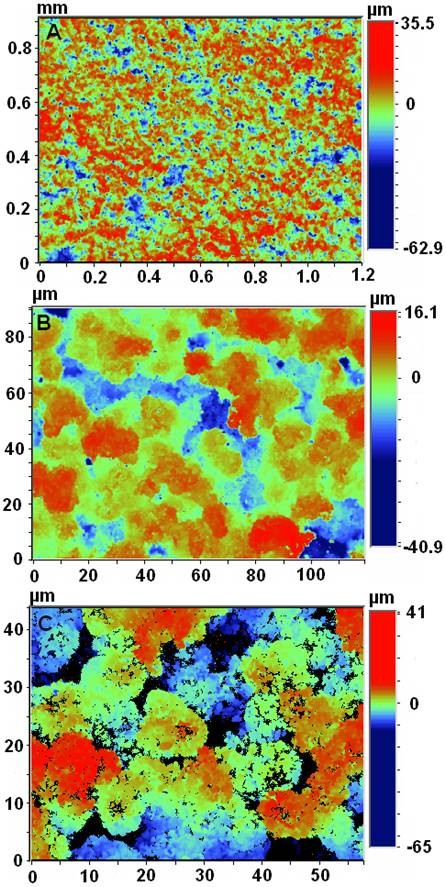
**Optical profiler images of the rough PLLA surface**. a, b and c are images taken with different magnifications.

**Figure 4:**
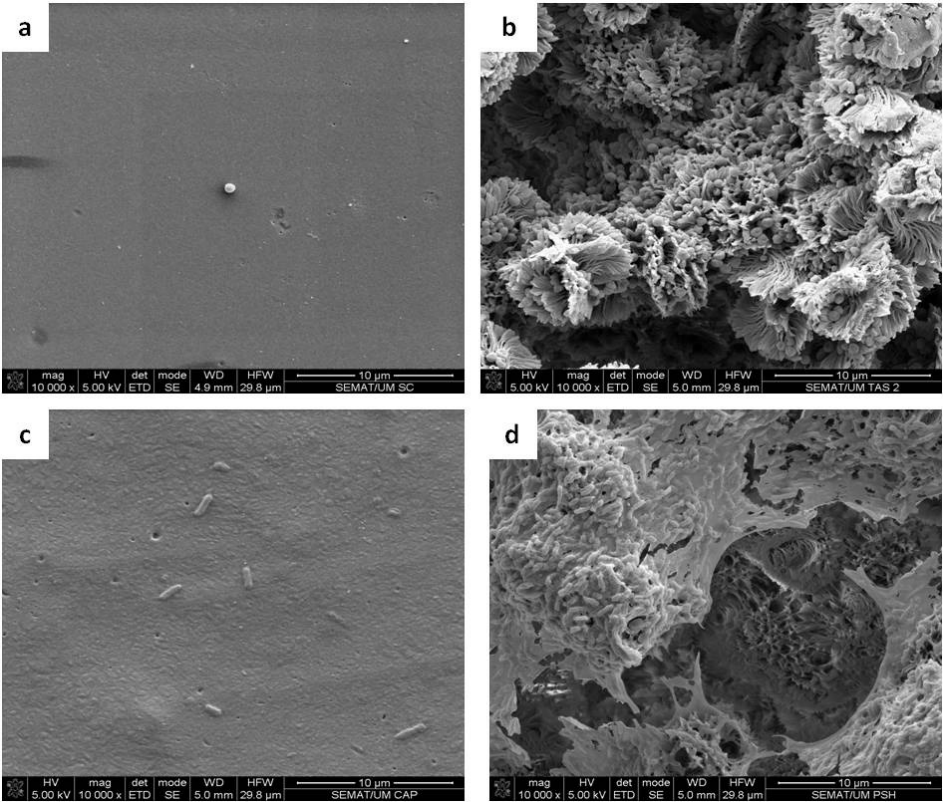
**SEM images showing *S. aureus *colonization of (a) H PLLA surface and (b) SH PLLA surface; and *P. aeruginosa *colonization of (c) H PLLA surface and (d) SH PLLA surface**.

SEM images, presented in Figure 2, show the contrasting morphologies of both surfaces tested, confirming that the H surface is much smoother (Figure 2a) than the SH surface, which is fully covered with papilla-like protrusions (Figures 2b and 2c) with sizes of about 10 μm. Such rough structure was also seen by optical profilometry (Figure 3) that indicated an average roughness of 8.28 μm and a diameter of each papillae of 8.97 μm, which is consistent with the size of such structures seen by SEM. It was also observed that, unlike *P. aeruginosa *cells, *S. aureus *cells seem to fit perfectly the holes and recesses on the SH surface (Figures 4a and 4b). Moreover, in contrast to what was observed for *S. aureus*, SEM images revealed that *P. aeruginosa *was able to produce biofilm on the SH surface, showing the presence of an extracellular matrix that, together with the cells, covered most of the rough surface (Figure 4c).

It is also important to note that the hydrophobicity of the same materials used in the present work had been previously assessed by contact angle (CA) measurements that showed a significant difference between both types of surfaces, with a CA mean value of 70° for the smooth H surface and 154° for the rough SH surface (Song et al. 2009).

## Discussion

Together with extracellular polymers and surface electrostatic charge, hydrophobicity is, undoubtedly, one of the critical surface properties (Gannon et al. 1991; Ahn and Lee 2003) since hydrophobic interactions define the strong attraction between hydrophobic molecules and surfaces in water. In biological systems, hydrophobic interactions are the strongest long-range non-covalent interactions and are considered a determining factor in microbial adhesion to surfaces (Sanin et al. 2003). Given the hydrophobic nature of the surfaces tested in the present study, the results obtained are in agreement with previous works, which show that *S. aureus *and *P. aeruginosa *preferentially colonize hydrophobic surfaces than hydrophilic ones (Ajayi et al. 2010; Zmantar et al. 2011). On the other hand, previous studies performed with the same SH surface used in the present work had demonstrated that almost no animal cell adhesion occurred (Alves et al. 2009; Song et al. 2009). These contrasting findings might be related with the accentuate differences between eukaryotic and prokaryotic cell walls, both in terms of morphology, length and surface properties. For instance, bacterial cells are about one tenth the size of animal cells, which enables them to fit into SH surface irregularities, while animal cells do not benefit from such a high contact surface. This is probably due to the fact that a rough surface has a greater surface area and the depressions in the roughened surfaces provide more favourable sites for colonization. Grooves or scratches that are in the order of bacterial size increase the contact area and hence the binding potential, whereas grooves that are much larger-wider than the bacterial size approach the binding potential of a flat surface. On the other hand, grooves or scratches too small for the bacterium to fit in them reduce the contact area of the bacterium and hence the binding ability (Edwards and Rutenberg 2001). The significant differences found regarding colonization of both surfaces by *S. aureus *(Figure 1) can be due to the combined effect of the different PLLA and *S. aureus *specific surface morphologies, since *S. aureus *cells seem to fit perfectly the irregularities on the SH surface (Figures 4a and 4b) and, thus, end up having a greater contact area than on the smooth H surface.

Concerning the colonization of the H surface, the significant differences found between bacterial strains (Figure 1) can be related with their distinct cell walls and extracellular polymeric substances (EPS). In fact, as a Gram-negative bacterium, the cell wall of *P. aeruginosa *contains lipids, proteins, and lipopolysaccharides (LPS), while the cell wall of the Gram-positive bacteria, such as *S. aureus*, does not contain LPS (Speranza et al. 2004). The LPS of *P. aeruginosa *are the major component of the outer surface, and are a well-established virulence factor (Fletcher et al. 1993; Rocchetta et al. 1999; Thuruthyil et al 2001), contributing to bacterial adhesion (Camesano and Logan 2000), most likely due to non-specific physiochemical interactions such as hydrophobicity (Thuruthyil et al. 2001). In this way, it is very likely that LPS present in *P. aeruginosa *cell wall are a determining factor in the colonization of the H surface, leading to a significant higher amount of cells in detriment to *S. aureus *(Figure 1). It is also described that the adhesion ability of *P. aeruginosa *is associated with the extensive production of EPS (Dunne 2002; Drenkard 2003). Thus, the high amount of EPS produced by *P. aeruginosa *might be responsible for biofilm formation on the SH surface (Figure 4d).

The results of the removal assays are in agreement with those found for the colonization assays, since a significant decrease of biomass of both bacterial strains was only found on the SH surface (Figure 1), suggesting, once again, that the distinct characteristics of both surfaces tested must be responsible for such outcomes. Thus, it is possible to infer that the crevices of the SH surface not only offer an increased area for attachment by providing more contact points, but also afford protection against shear forces (Verran and Boyd 2001; Whitehead and Verran 2006). Moreover, the extracellular matrix formed by *P. aeruginosa *cells on the SH material might also had a protective effect during the removal assays, due to its crucial role in maintaining structural integrity of *P. aeruginosa *biofilms (Chen and Stewart 2002).

In conclusion, this work showed that both PLLA surfaces tested are able to be colonized by bacterial cells, regardless of their Gram-type and morphology. Nevertheless, a further analysis comparing the results obtained with both surfaces revealed that SH PLLA supported a higher amount of *S. aureus *cells, enabled biofilm formation by *P. aeruginosa *cells, and also suffered less bacteria removal when compared to the H surface. Therefore, it can be said that SH surfaces are not suitable for biomedical applications with antimicrobial properties. Conversely, this work introduces a possible application of PLLA-based superhydrophobic materials as bacterial colonization substrata with potential to be used as carriers for biomass immobilization in bio-reactors. Nevertheless, these studies are yet preliminary, since a higher number of strains need to be tested in order to address the intra-species variability in terms of surface characteristics and their consequent interaction with these surfaces. Likewise, a wider range of bacterial species, as well as other microorganisms with biotechnological potential, such as yeasts, and experimental conditions (culture media, temperature, incubation period, shear force, etc), need to be studied to confirm the conclusions presented here, and to clarify the observed high potential of using such modified surfaces as microbial colonization substrata in biotechnological processes.

## Competing interests

The authors declare that they have no competing interests.
